# Object-grounded embodied picking for e-commerce warehouse fulfillment: a foveated diffusion policy for operational robustness

**DOI:** 10.3389/frobt.2026.1883320

**Published:** 2026-07-30

**Authors:** Chaoqun Li, Longfei Wang, Huiying Xu, Xiaolei Zhang, Zhenglong Wan, Xinzhong Zhu

**Affiliations:** 1 School of Education and Management, Bozhou Vocational and Technical College, Bozhou, Anhui, China; 2 School of Computer Science and Technology, Zhejiang Normal University, Jinhua, China; 3 Hefei Geek+ Robotics Co., Ltd., Hefei, China

**Keywords:** e-commerce warehouse fulfillment, embodied picking, foveated diffusion policy, object-grounded visuomotor learning, operational robustness

## Abstract

Embodied picking for e-commerce fulfillment remains vulnerable to dense clutter, reflective packaging, and background variation, which can undermine the effectiveness and robustness of visuomotor policies learned from demonstrations. A key limitation is the absence of explicit object grounding, causing policies to exploit spurious contextual cues rather than task-relevant visual evidence. To address this issue, we propose the Foveated Diffusion Policy (FDP), which integrates object-centric visual grounding into diffusion-based action generation. FDP adopts a panorama–fovea dual-stream visual encoder: a low-resolution panorama stream captures global scene context, while a detection-guided fovea stream uses ROI Align to extract high-resolution object tokens. At each denoising step, these tokens are incorporated into the diffusion denoiser through object-guided cross-attention, thereby grounding action generation in target-specific visual evidence instead of undifferentiated global representations. We evaluate FDP on ten benchmark datasets spanning six simulated manipulation tasks with both proficient-human and multi-human demonstrations. FDP achieves average success rates of 81.0% and 76.8%, respectively, outperforming diffusion-based baselines and delivering particularly strong performance on object-centric tasks, including Push-T (86.7 ± 2.5%) and Lift (100.0 ± 0.0%). Ablation studies on a diagnostic subset show that foveated conditioning improves the average success rate by 11.3 percentage points over the non-foveated variant. FDP also maintains an 84.0% success rate under severe localization perturbations (σ = 10 pixels), demonstrating resilience to detection noise. Furthermore, accelerated DDIM sampling reduces policy-inference latency to approximately 55 ms with 10 denoising steps, supporting the feasibility of closed-loop control. Collectively, these results demonstrate that explicit object grounding is a promising and computationally practical approach to improving the robustness of visuomotor policies in warehouse-relevant simulated manipulation scenarios.

## 1 Introduction

E-commerce and omnichannel fulfillment have substantially increased the operational demands placed on warehouse picking systems. In contemporary warehouse environments, embodied picking agents are expected to retrieve target items from bins or conveyor-fed workspaces under dense clutter, frequent occlusion, reflective packaging, and changing illumination or background conditions. These challenges are further intensified by SKU heterogeneity, rapid inventory turnover, and continuously changing stacking patterns, which introduce persistent visual and operational disturbances into fulfillment processes. Consequently, a substantial gap remains between laboratory-level manipulation performance and the robustness required for deployment-oriented warehouse fulfillment. This gap is reflected in long-standing warehouse-picking challenges as well as in recent datasets and benchmarks designed to evaluate robotic perception and manipulation under cluttered, reflective, and visually complex conditions (Correll et al., 2018; Yang et al., 2021; D’Avella et al., 2024).

Traditional warehouse-picking systems often rely on explicit geometric perception, including 6D pose estimation, model-based matching, and RGB-D perception. Benchmarks like BOP and pose-estimation methods such as PoseCNN and DenseFusion (Hodan et al., 2018; Xiang et al., 2017; Wang et al., 2019) have advanced this line of research. Although geometric perception can be effective under structured conditions, its deployment may become fragile or costly when depth sensing is unreliable, objects are transparent or reflective, or item configurations change frequently. These limitations are further highlighted by datasets and benchmarks focusing on reflective, transparent, or cluttered objects, such as ROBI and ClearPose (Yang et al., 2021; Chen et al., 2022). For e-commerce fulfillment, these observations indicate a practical requirement: embodied picking systems must remain reliable under imperfect localization, visual variation, and high-throughput operational constraints.

Recent advances in imitation learning have improved the learning of end-to-end visuomotor policies from demonstrations. Under the behavioral cloning paradigm and its extensions, policies can map raw observations directly to actions and have shown promising performance in robotic manipulation tasks (Levine et al., 2016). Covariate shift compensation techniques further enhance the policy stability in sequential decision-making (Ross et al., 2011). To overcome the unimodal action regression constraint, implicit, energy-based, and transformer-based formulations are proposed (for better multimodal action distribution modeling in manipulation tasks Florence et al. (2022); Finn et al. (2016); Muhammad et al. (2022). Recently, generative policy families like action-chunking transformers and diffusion-based policy learning have enhanced action expressiveness and temporal consistency by modeling multimodal action trajectories rather than predicting an average action (Zhao et al., 2023; Chi et al., 2024; Ho et al., 2020). Planning-focused diffusion methods demonstrate the potential of diffusion models to guide sequential decision-making Janner et al., 2022).

Despite these advances, most existing policy-learning methods primarily strengthen how actions are generated, while paying less attention to whether the visual evidence conditioning those actions remains anchored to the intended target object. This limitation is particularly consequential in warehouse fulfillment scenarios, where observations contain abundant nuisance variation, including background textures, specular highlights, transient distractors, and visually similar neighboring items. Under such conditions, end-to-end policy learning may become overly dependent on spurious contextual correlations that coincide with successful demonstrations but are not intrinsically related to correct target interaction. This failure mode is closely related to shortcut learning, in which models exploit superficial statistical regularities that do not generalize under distribution shift (Geirhos et al., 2020). In warehouse-like environments, such behavior can appear as visual myopia: the policy may respond to irrelevant contextual cues rather than to the geometry and location of the target object, leading to brittle failures under changes in lighting, background configuration, clutter arrangement, or packaging appearance.

A complementary line of research improves manipulation by introducing more structured visual representations. Object-centric learning methods, such as Slot Attention, decompose visual scenes into object-level constituents and support compositional reasoning (Locatello et al., 2020). In robotic manipulation, spatially structured architectures such as Transporter Networks and dual-pathway designs such as CLIPort combine semantic understanding with spatially precise action prediction (Zeng et al., 2021; Shridhar et al., 2022). In parallel, large-scale visual pretraining methods such as R3M and VIP improve transferability and data efficiency in downstream manipulation tasks (Nair et al., 2023; Ma et al., 2023). Attention-based robustness studies further suggest that emphasizing task-relevant regions can improve policy resilience under visual disturbance (Abolghasemi et al., 2019). Nevertheless, these approaches are often designed for general representation transfer, spatial action prediction, or one-stage feature selection. They do not directly address how target-object evidence should be repeatedly retrieved and incorporated into a generative control process under cluttered and visually unstable warehouse-like conditions.

For embodied picking in e-commerce warehouse fulfillment, the central challenge is therefore not only to generate multimodal actions, but also to preserve target-grounded perception throughout action generation. Robust fulfillment-oriented picking requires the policy to distinguish scene-level constraints from object-level evidence and to prevent generated actions from drifting toward irrelevant background cues. From an operational perspective, this issue is critical because visually induced action instability may increase retry behavior, cycle-time variability, manual intervention, and exception-handling costs. Existing studies thus reveal a common limitation: action models have become increasingly expressive, and perception models have become more structured, yet target-grounded visual evidence remains insufficiently integrated into policy generation for warehouse-relevant embodied picking.

**FIGURE 1:**
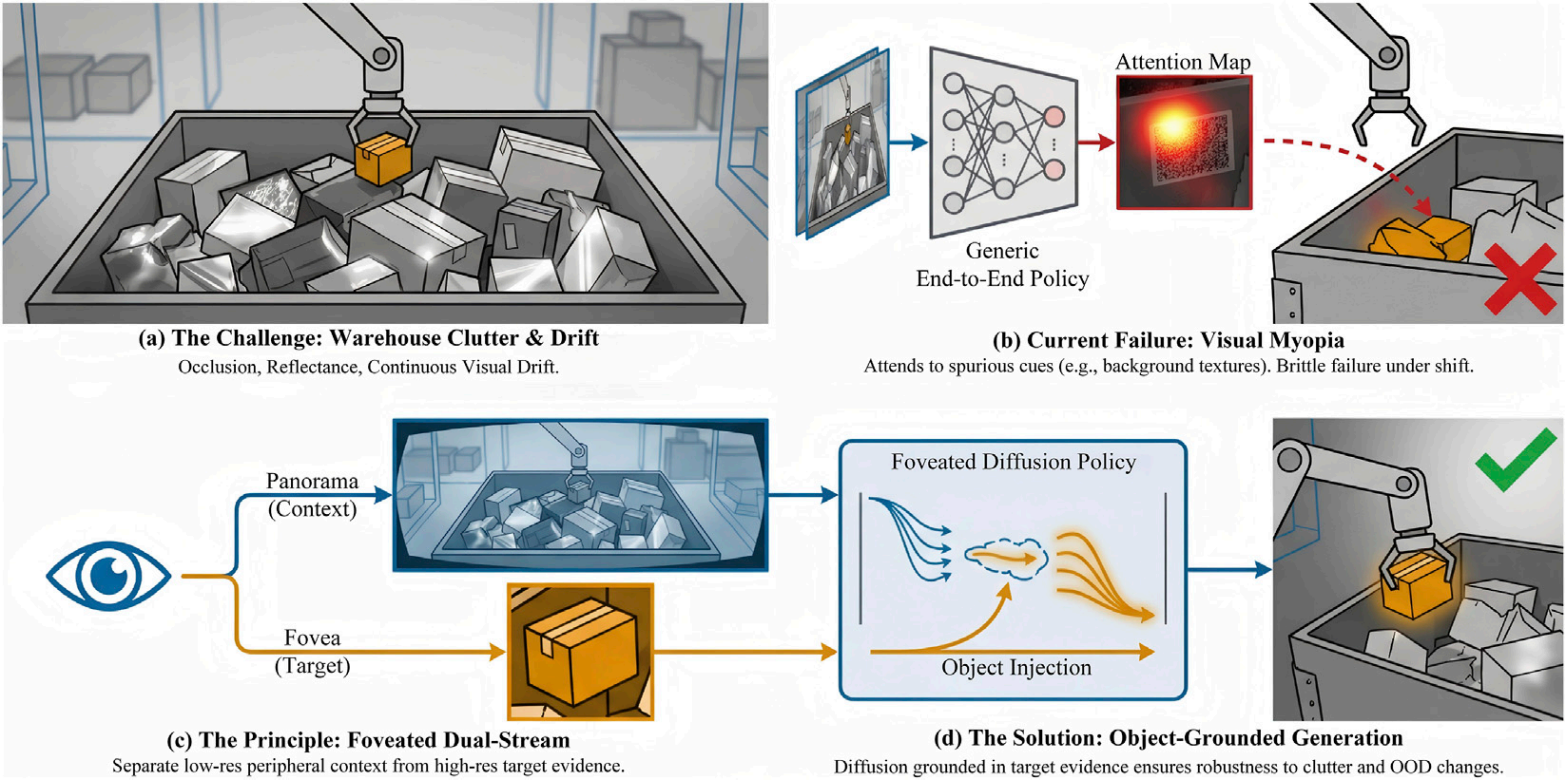
Object-Grounded Embodied Picking for Operational Robustness in E-Commerce Warehouse Fulfillment. **(a)** Warehouse fulfillment scenes are characterized by cluttered item arrangements, reflective packaging, occlusion, and changing visual backgrounds. **(b)** Under such disturbances, generic end-to-end policies may rely on spurious context, resulting in unstable target selection and poor robustness under visual shifts. **(c)** FDP separates peripheral scene context from target-centered high-resolution evidence through a foveated dual-stream perception design. **(d)** This object-grounded generation mechanism provides a structured route toward more robust embodied picking under warehouse-relevant visual disturbance and imperfect object localization.

To address this problem, this study proposes a Foveated Diffusion Policy (FDP) for object-grounded embodied picking, as illustrated in Figure 1. The core idea is to separate object-centric evidence from scene context and inject this structured perception into diffusion-based action generation. Inspired by findings from natural visual behavior showing that task execution depends on selective focus on relevant targets while preserving peripheral scene awareness (Hayhoe and Ballard, 2005), FDP introduces a panorama-fovea dual-stream perception framework. Specifically, a context stream encodes low-resolution global scene layout and workspace constraints, while a detection-guided fovea stream extracts high-resolution object features from target-centered regions. These object features are incorporated into the diffusion denoising process through an object-guided cross-attention mechanism, enabling the policy to repeatedly retrieve task-relevant target evidence during action generation. In this way, FDP strengthens object-grounded visuomotor learning for warehouse-relevant embodied picking, rather than relying solely on undifferentiated global visual representations or object-centric features that are not explicitly coupled with generative action refinement (Locatello et al., 2020).

We summarize our contributions as three verifiable claims:

1. A foveated dual-stream visual encoder for warehouse-relevant embodied picking. We develop a panorama-fovea dual-stream encoder that separates global scene context from target-centered object evidence, thereby helping reduce representational confusion caused by clutter, packaging reflectance, and background variation.
2. An object-guided diffusion denoising mechanism for target-conditioned action generation. We incorporate object-guided cross-attention into the diffusion transformer backbone, enabling each denoising step to retrieve relevant object tokens and strengthening the grounding of generated actions in task-relevant target evidence.
3. A detector-tolerant and low-latency policy inference pipeline for deployment-relevant robustness. By combining bounding-box perturbation analysis, box-jittering regularization, and accelerated DDIM (Song et al., 2021) sampling, we examine the tolerance of FDP to imperfect localization while supporting low-latency policy inference under warehouse-relevant manipulation settings.

## 2 Methodology

**FIGURE 2:**
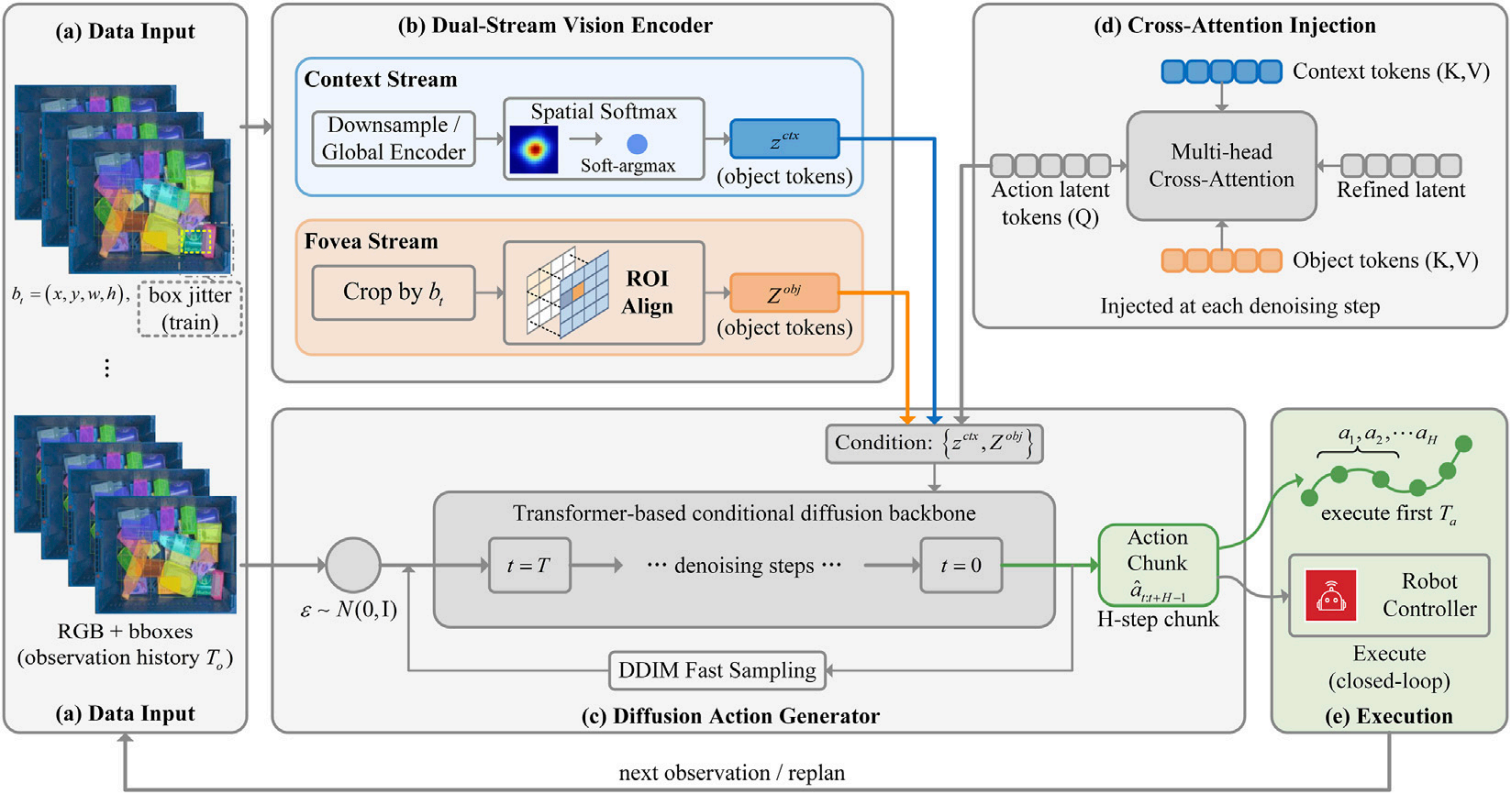
Overall Architecture of Foveated Diffusion Policy. **(a)** The system takes an RGB observation history and the corresponding bounding boxes as input. **(b)** The dual-stream vision encoder separates full-scene context from target-centered object evidence. **(c)** A transformer-based conditional diffusion model generates an action chunk from the visual conditioning signal. **(d)** Multi-head cross-attention injects visual tokens into the denoising process. **(e)** The first $T_{a}$ actions are executed in a closed loop, and the policy replans at the next control cycle.

This section presents the proposed FDP for object-grounded embodied picking in warehouse-relevant fulfillment settings. The method is designed to improve robustness under clutter, reflective packaging, occlusion, and scene variation by separating target-centered object evidence from scene-level context and incorporating this distinction into diffusion-based action generation. As shown in Figure 2, FDP consists of a dual-stream visual encoder, a conditional diffusion action generator, and an object-guided cross-attention module. Compared with generic end-to-end visuomotor policies, the key difference lies in the conditioning pathway: scene-level context preserves workspace layout and boundary constraints, whereas target-centered object tokens provide task-relevant evidence for action refinement.

### 2.1 System overview

We consider visuomotor imitation learning from demonstrations collected in cluttered manipulation scenes. Each sample contains an observation history, a future action chunk, and the corresponding target bounding boxes:

$$D=\left\{\left(o_{t-T_{o}+1:t},\;a_{t:t+H-1},\;b_{t-T_{o}+1:t}\right)\right\},$$ (1)

where $T_{o}$ is the observation horizon, H is the action horizon, $o_{t-T_{o}+1:t}$ denotes the RGB observation sequence, $a_{t:t+H-1}\in R^{H\times d_{a}}$ is a continuous action chunk, and $b_{t-T_{o}+1:t}$ denotes the 2D bounding boxes with validity flags. The policy predicts an action chunk from recent observations and target-localization cues:

$$\pi_{\theta }:\left(o_{t-T_{o}+1:t},\;b_{t-T_{o}+1:t}\right)\mapsto {\hat{a}}_{t:t+H-1}.$$ (2)

To maintain closed-loop stability, only the first $T_{a}$ actions are executed at each control cycle,

$${\hat{a}}_{t:t+T_{a}-1}^{\text{exec}}={\hat{a}}_{t:t+T_{a}-1},\;T_{a}\leq H,$$ (3)

after which, the policy replans using updated observations. This receding-horizon strategy follows the action-chunking logic of Diffusion Policy (Chi et al., 2024), while FDP differs in how visual conditioning is constructed. Specifically, the full image is encoded as scene context, target-centered regions are encoded as object tokens, and both are injected into the diffusion model during denoising. The system therefore proceeds through four stages: dual-stream visual encoding, diffusion-based action generation, object-guided cross-attention injection, and closed-loop execution. This design allows the policy to retain global workspace awareness while grounding action refinement in target-relevant evidence.

### 2.2 Diffusion for visuomotor control

**FIGURE 3:**
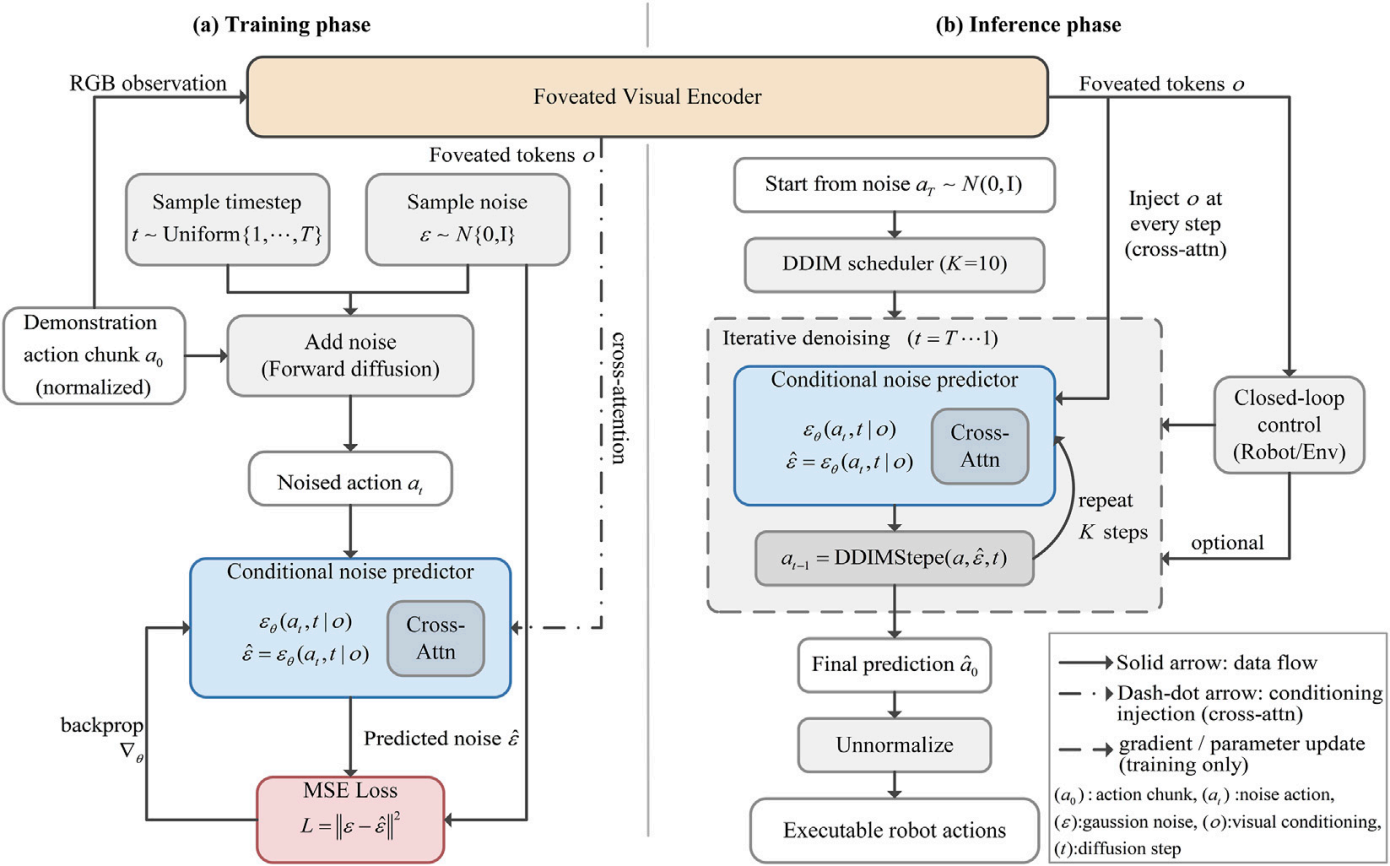
Training and Inference Flow of Diffusion Action Control. **(a)** Training phase: Gaussian noise is added to the demonstration action chunk at a randomly sampled diffusion step, and the conditional network predicts the injected noise under visual conditioning. The model is trained by minimizing the mean-squared error between the predicted and true noise. **(b)** Inference Phase: the process starts from Gaussian noise and iteratively denoises for a fixed number of DDIM steps. The conditioning signal is injected at every denoising step via cross-attention, and the final prediction is converted into executable robot actions for closed-loop control.

We model embodied picking as conditional action-chunk generation using denoising diffusion probabilistic models (DDPM), following the basic formulation of Diffusion Policy (Chi et al., 2024; Ho et al., 2020). The prediction target is a short-horizon action chunk, and the conditioning signal is produced by the dual-stream visual encoder. Let $X_{0}=a_{t:t+H-1}\in R^{H\times d_{a}}$ denote a continuous action sequence with a horizon length of H , where each action possesses a dimensionality of $d_{a}$ . The vector c serves as the conditioning signal. As shown in Figure 3, in FDP, the conditioning signal is organized as:

$$c=\left(z^{\text{ctx}},\;Z^{\text{obj}}\right),$$ (4)

where $z^{\text{ctx}}$ is the scene-level context feature and $Z^{\text{obj}}$ is the set of object-centered features.

For the forward process, we adopt the standard variance-preserving diffusion formulation. Given a predefined noise schedule ${\left\{\beta_{k}\right\}}_{k=1}^{K}$ , where $\beta_{k}\in \left(0,1\right)$ , we define $\alpha_{k}=1-\beta_{k}$ , ${\bar{\alpha }}_{k}=\prod_{i=1}^{k}\alpha_{i}$ . The forward transition is:

$$q\left(X_{k}|X_{k-1}\right)=N\left(\sqrt{\alpha_{k}}X_{k-1},\;\left(1-\alpha_{k}\right)I\right),\;k=1,\ldots ,K.$$ (5)

The marginal distribution of $X_{k}$ conditioned on the clean action $X_{0}$ is:

$$q\left(X_{k}|X_{0}\right)=N\left(\sqrt{{\bar{\alpha }}_{k}}X_{0},\;\left(1-{\bar{\alpha }}_{k}\right)I\right),$$ (6)

which is equivalently sampled by:

$$X_{k}=\sqrt{{\bar{\alpha }}_{k}}X_{0}+\sqrt{1-{\bar{\alpha }}_{k}}\epsilon ,\;\epsilon ∼N\left(0,I\right).$$ (7)

During training, a diffusion step k and a Gaussian noise sample ϵ are randomly drawn, and the scheduler is used to generate the corresponding noised action $X_{k}$ . A conditional noise-prediction network $\epsilon_{\theta }$ then estimates the injected noise under conditioning c . The model is optimized using the standard noise-prediction objective:

$$L=E_{k,X_{0},c,\epsilon }\left[{\left\|\epsilon -\epsilon_{\theta }\left(\sqrt{{\bar{\alpha }}_{k}}X_{0}+\sqrt{1-{\bar{\alpha }}_{k}}\epsilon ,\;k,\;c\right)\right\|}_{2}^{2}\right].$$ (8)

At inference time, the action sequence is initialized from Gaussian noise and iteratively denoised to obtain the final action prediction. The generic reverse update at step k is written as:

$$X_{k-1}=\alpha \left(k\right)\left(X_{k}-\gamma \left(k\right)\epsilon_{\theta }\left(X_{k},k,c\right)\right)+\sigma \left(k\right)\eta ,\;\eta ∼N\left(0,I\right).$$ (9)

where the α(k) , γ(k) , and σ(k) are determined by the chosen noise schedule.

Because closed-loop robot control is sensitive to delay, we use DDIM sampling at inference and limit the number of denoising steps to 10. After denoising, only the first $T_{a}$ actions are executed, and the policy replans at the next control cycle. This receding-horizon design is consistent with diffusion-based action chunking, while the key difference of FDP lies in its visual conditioning: the denoising process is guided jointly by scene-level context and target-centered object evidence, enabling more stable action refinement in cluttered warehouse-relevant environments.

### 2.3 Dual-stream vision encoder

**FIGURE 4:**
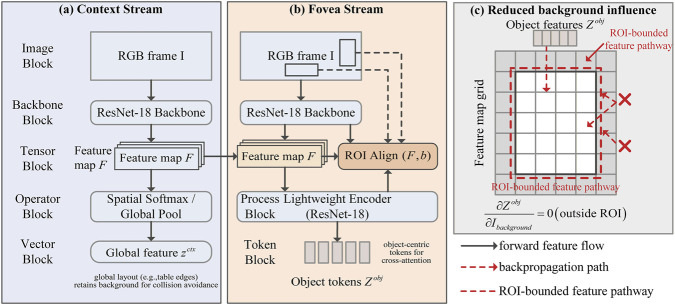
Dual-Stream Vision Encoder. **(a)** The context stream processes the full image to extract global spatial features that preserve scene-level constraints. **(b)** The fovea stream applies ROI Align based on bounding boxes to obtain target-centered object tokens. **(c)** Compared with single-stream encoding, the ROI-bounded branch reduces the influence of out-of-ROI background regions on object-feature learning.

In cluttered warehouse-relevant scenes, a single-stream visual encoder may mix target geometry with background texture, reflectance, and stacking artifacts, which weakens robustness under visual disturbance. To address this issue, as depicted in Figure 4, FDP employs a dual-stream encoder that separates scene-level context from target-centered evidence through two complementary branches. The context stream preserves global layout constraints from the full image, whereas the fovea stream applies ROI Align around detected bounding boxes to extract target-centered object tokens. This design introduces a more target-focused conditioning pathway and reduces the influence of irrelevant background content on object-feature learning.

For the context stream, we apply a context encoder to the full image sequence:

$$z^{\text{ctx}}=f_{\text{ctx}}\left(I_{t-T_{o}+1:t}\right)\in R^{T_{o}\times D_{\text{ctx}}}.$$ (10)

In implementation, we use ResNet-18 [27] as the backbone and apply spatial softmax across the spatial dimensions to generate a compact representation for each frame. This branch preserves scene-level information such as workspace boundaries, obstacles, and free-space structure, which remains necessary for feasible manipulation and collision avoidance.

In contrast, the fovea stream restricts direct feature extraction to ROI-bounded target regions and reduces, rather than eliminates, background interference. For each frame, ROI Align is applied to extract target-centered patches, which are then encoded into object tokens:

$$Z^{\text{obj}}=f_{\text{obj}}\left(I_{t-T_{o}+1:t},\;b_{t-T_{o}+1:t}\right)\in R^{T_{o}\times N\times D_{\text{obj}}}.$$ (11)

Here, N represents the number of items found in each frame, and the $D_{\text{obj}}$ is matched to the main feature dimension so that these tokens can be used directly as keys and values in the subsequent cross-attention module. Compared with full-image encoding, this branch concentrates feature learning on the target region and reduces interference from irrelevant background content.

The distinction between the two streams is also reflected in the gradient pathway. In a single-stream encoder:

$$f=Enc\left(I\right),\;\frac{\partial f}{\partial I\left[background\right]}\neq 0.$$ (12)

which indicates that background regions can influence the global representation through shared convolutional filters. In contrast, the fovea branch first restricts feature computation to ROI-centered regions and then encodes the cropped patches:

$$f^{\text{obj}}=Enc\left(ROIAlign\left(F\left(I\right),\;b\right)\right).$$ (13)

where Equation 13 should be understood as an idealized description of the ROI-bounded branch rather than as a claim of complete background independence. In practice, the fovea stream restricts direct feature computation to target-centered regions and thereby reduces the influence of out-of-ROI background pixels on object-token learning. The role of this design is to weaken background shortcuts, not to establish strict causal isolation between object and background signals.

The proposed dual-stream design differs from existing visual-focusing mechanisms in three ways. The fovea stream applies ROI Align to externally detected bounding boxes, so its localization is exogenous and instance-grounded. Soft attention instead learns attention weights from the feature map, and a spatial transformer network regresses one global affine warp; neither uses explicit object localization. The policy also keeps an independent context stream that the fovea stream never re-weights or warps. As shown in Equations 12, 13, a single shared feature map lets background regions influence the representation, so Soft Attention and STN, which operate on one entangled map, have no detection-independent context pathway. Finally, object tokens are injected as keys and values into cross-attention at every denoising layer, rather than performing one-stage feature selection or global modulation such as FiLM.

### 2.4 Object-guided injection via cross attention fusion

**FIGURE 5:**
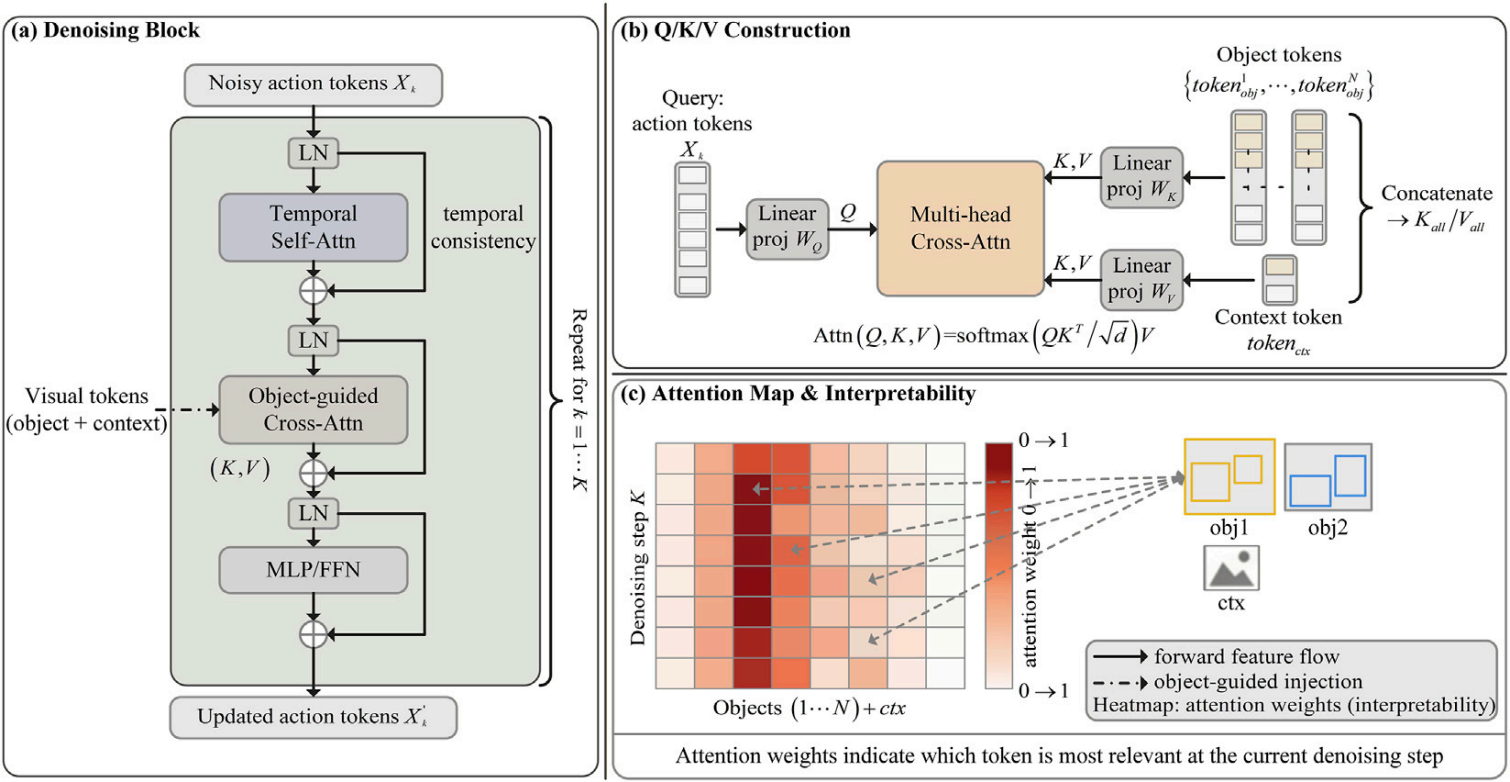
Object-guided Cross-Attention Injection Mechanism. **(a)** Each denoising block contains self-attention, object-guided cross-attention, and an MLP. **(b)** Noisy action tokens serve as queries, and the concatenated visual tokens serve as keys and values. **(c)** The attention map provides an interpretable view of which object or context token is most relevant to the current denoising step during action generation.

Simply separating object and context features at the visual front end is not sufficient if the action generator does not use this distinction during denoising. FDP therefore injects object evidence into every denoising layer of the diffusion Transformer through object-guided cross-attention. As depicted in Figure 5, the module first flattens object features, optionally appends a context token, and then performs cross-attention between noisy action tokens and visual tokens. In this way, denoising is repeatedly guided by target-relevant visual evidence rather than relying only on a single global latent representation.

Let the dual-stream encoder output context features $z^{\text{ctx}}\in R^{B\times T_{o}\times D_{\text{ctx}}}$ and object features $Z^{\text{obj}}\in R^{B\times T_{o}\times N\times D_{\text{obj}}}$ for a batch size B , observation horizon $T_{o}$ , and up to N objects per frame. The object features are first flattened across time and instances to form the token sequence:

$$Z_{\text{flat}}^{\text{obj}}=Flatten\left(Z^{\text{obj}}\right)\in R^{B\times \left(T_{o}N\right)\times D_{\text{obj}}}.$$ (14)

The context feature is then projected, if necessary, and appended as an additional token:

$${\tilde{z}}^{\text{ctx}}=Pool\left(z^{\text{ctx}}\right)\in R^{B\times 1\times D}.$$ (15)

where, if $D_{\text{ctx}}\neq D_{\text{obj}}$ , linear layers or MLPs are used to project both streams into a common dimension D . The final visual token sequence is then constructed as:

$$Z^{\text{vis}}=Concat\left(Proj\left(Z_{\text{flat}}^{\text{obj}}\right),{\tilde{z}}^{\text{ctx}}\right)\in R^{B\times \left(T_{o}N+1\right)\times D}.$$ (16)

At the diffusion step k , let the latent representation of the noisy action chunk be $X_{k}\in R^{B\times H\times d_{a}}$ is first embedded into an action-latent sequence:

$$h_{k}=Embed\left(X_{k},k\right)\in R^{B\times H\times D}.$$ (17)

Queries, keys, and values are defined by learned linear projections:

$$Q=h_{k}W_{Q},\;K=Z^{\text{vis}}W_{K},\;V=Z^{\text{vis}}W_{V},$$ (18)

and multi-head cross-attention [28] is computed as:

$$Attn\left(Q,K,V\right)=softmax\left(\frac{QK^{⊤}}{\sqrt{d_{h}}}\right)V,$$ (19)

where $d_{h}$ denotes the dimensionality of each attention head. Within each transformer block, self-attention captures temporal dependencies within the action-token sequence, whereas cross-attention retrieves task-relevant visual evidence from the object-context token set:

$$\begin{array}{cc} h & \leftarrow h+SelfAttn\left(h\right),\\ h & \leftarrow h+CrossAttn\left(h,K_{\text{all}},V_{\text{all}}\right),\\ h & \leftarrow h+FFN\left(h\right). \end{array}$$ (20)

Compared with global modulation schemes such as FiLM [29], cross-attention provides a more explicit mechanism for conditioning action refinement on visual tokens. The attention matrix offers a descriptive view of how object and context tokens are retrieved during denoising. Tokens receiving higher attention weights can be interpreted as being more strongly associated with the current action refinement step, but these weights should be treated as interpretability cues rather than causal evidence.

## 3 Experiments and analysis

### 3.1 Experiment settings

In this study, all experiments are conducted on a dedicated workstation running Ubuntu 24.04 LTS on an x86_64 architecture. The workstation is equipped with four NVIDIA RTX 4090 GPUs. Unless otherwise specified, each training run is executed on a single GPU, while multiple GPUs are used to parallelize tasks, seeds, and benchmark settings. The hardware and software environments are summarized in Table 1.

**TABLE 1:** Experimental environment.

(a) Hardware configuration		(b) Software environment	
Component	Specification	Package	Version
GPU	NVIDIA RTX 4090×4 (24 GB VRAM each)	Operating system	Ubuntu 24.04 LTS
CPU	Intel xeon gold 6,133 (40 cores)	Python	3.9
System memory	256 GB DDR4	PyTorch	2.4.0 (CUDA 12.1)
		Diffusers	0.35.2
		Robomimic	0.2.0
		MuJoCo	3.3.7

#### 3.1.1 Data preparation and curation

We evaluate FDP on ten simulated manipulation benchmark settings that are relevant to warehouse fulfillment and cover a broad range of task difficulty. The benchmark suite is drawn from two established sources: Push-T (Florence et al., 2022; Mandlekar et al., 2022). The ten settings consist of one Push-T benchmark, four Robomimic tasks evaluated under both proficient-human (ph) and multi-human (mh) demonstrations, and one ToolHang benchmark. Following standard protocols, ph demonstrations represent relatively consistent expert behavior, whereas mh demonstrations contain greater behavioral diversity across operators.

As summarized in Table 2, the resulting tasks span planar manipulation, single-arm manipulation, bimanual coordination, and precision manipulation. Although these datasets are simulated rather than collected from operational e-commerce warehouses, they capture perception-control difficulties closely related to warehouse fulfillment, including cluttered object placement, target localization under visual variation, multi-view coordination, and fine-grained insertion or placement. They therefore provide a controlled benchmark suite for evaluating whether object-grounded embodied picking can improve robustness under warehouse-relevant visual and operational disturbances.

**TABLE 2:** Overview of warehouse-relevant simulated manipulation benchmarks.

Task	Category	Episodes	Action dim	Cameras	Key challenge	Fulfillment analogue
Push-T	Planar	206	2	1	Non-prehensile manipulation	Cluttered item singulation/pushing items apart
Lift	Single-arm	200/300	7	2	Basic grasping	Bin picking of individual packaged items
Can	Single-arm	200/300	7	2	Precise placement	Item placement into tote or sorting zone
Square	Single-arm	200/300	7	2	Alignment under occlusion	Alignment-sensitive handling under occlusion
Transport	Bimanual	200/300	14	4	Coordination across arms	Handoff or multi-station transfer
ToolHang	Precision	200	7	2	Fine-grained insertion	Handling irregular or hanging items

Tasks are grouped by manipulation complexity, with demonstration quality indicated by the suffixes ph (proficient-human) and mh (multi-human).

As a preprocessing step, all datasets are augmented with object-centric bounding-box annotations. Grounding DINO Liu et al., 2024 is used with task-specific prompts to detect manipulated objects frame by frame, and the resulting detections are temporally associated where needed to form bounding-box sequences. Each sample is augmented with normalized bounding-box coordinates and validity masks. These annotations provide the target-localization cues required by the foveated branch of FDP. Since bounding-box information constitutes an explicit structural prior for the proposed method, its contribution is examined through ablation and perturbation studies reported later.

#### 3.1.2 Model parameters

FDP combines a transformer-based diffusion denoiser with a dual-stream visual encoder. The context stream uses a ResNet-18 backbone to encode full-scene observations into compact scene-level features, while the fovea stream encodes target-centered regions into object tokens. These complementary representations are fused through object-guided cross-attention during denoising. Table 3 summarizes the main architectural and optimization settings used in the experiments.

**TABLE 3:** Core hyperparameters for foveated diffusion policy.

Component	Parameter	Value
Temporal window	$T_{o}$ / $T_{p}$ / $T_{a}$ / $T_{l}$	2/16/8/0
Transformer	Layers/Heads/Dim	8/4/256
	Attention dropout	0.01 (Push-T), 0.3 (Robomimic)
Vision encoder	Backbone	ResNet-18 (ImageNet pretrained)
	Feature dimension	512→256 (projected)
Foveated attention	Max objects	2–3
	Fovea scale factor	1.5×
Diffusion	Training diffusion steps	100
	Default inference steps	10 (DDIM)
	Noise schedule	Squared-cosine
Optimization	Optimizer	AdamW ( $lr=1\times 10^{-4}$ , $wd=1\times 10^{-3}$ )
	Schedule	Cosine annealing (1000-step warmup)
	Epochs/EMA decay	3,050/0.9999

All models are trained for 3,050 epochs using AdamW with cosine annealing and a 1000-step linear warmup. Batch sizes are selected according to memory requirements: 64 for standard single-arm tasks, 48 for the multi-camera Transport task, and 24 for the high-resolution ToolHang task. On a single NVIDIA RTX 4090, training time ranges from approximately 40–93 h for standard benchmarks and 250–500 h for Transport and ToolHang. Because the method is intended for closed-loop control, the default inference configuration uses DDIM with 10 denoising steps, consistent with the deployment-oriented setting introduced in Section 2. Latency under reduced DDIM sampling steps is analyzed separately in Section 3.2.3.

#### 3.1.3 Evaluation protocol

We use success rate (SR) as the primary evaluation metric. SR is defined as the proportion of rollout episodes that satisfy the task-specific success criterion within the allowed horizon. For Push-T, success is defined as achieving at least 95% coverage between the T-block and the target region. For Robomimic tasks, success is determined by the simulator-provided binary task indicator. The task-specific rollout limits and the number of evaluation episodes are listed in Table 4.

**TABLE 4:** Task-specific success criteria and evaluation parameters.

Task	Success criterion	Max steps		Eval episodes
		ph	mh	
Push-T	Coverage ≥95%	300	–	50
Lift	Object at target height	400	500	50
Can	Can be placed in the target zone	400	500	50
Square	Nut aligned with the peg slot	400	500	50
Transport	Object transferred between arms	700	700	50
ToolHang	Tool inserted into the hook	700	–	50

All experiments are repeated with three random seeds (42, 43, and 44), each indexing an independent training run with a distinct network initialization and stochastic optimization trajectory. Because a seed only initializes the pseudo-random number generator state, the numerical proximity of the seed values does not affect the statistical independence of the runs or the run-to-run variance being estimated; the same seed set is applied identically to all baselines and ablations to keep comparisons unbiased. Results are reported as mean ± standard deviation. Evaluation rollouts are performed every 50 training epochs, and the final reported checkpoint is selected according to validation-style rollout performance using the same selection rule for all methods. In addition to SR, later experiments assess robustness through three complementary analyses: sensitivity to bounding-box perturbation, performance under multi-human demonstration diversity, and inference latency under reduced DDIM sampling steps. These secondary analyses support the paper’s robustness claims but do not replace the primary task-success metric.

#### 3.1.4 Baseline methods

We compare FDP with four policy-level baselines covering recurrent behavioral cloning, action-chunking transformer policies, and diffusion-based visuomotor policies, as summarized in Table 5. These baselines are selected to represent both conventional imitation-learning models and recent generative action-generation frameworks under aligned observation inputs and comparable training protocols. To avoid conflating policy formulation with sampling strategy, DDPM- and DDIM-based sampling configurations are not treated as separate policy baselines in the main comparison. Instead, inference efficiency under reduced DDIM steps is analyzed separately in Section 3.2.3.

**TABLE 5:** Baseline methods and their characteristics.

Method	Paradigm	Action modeling
LSTM-GMM Mandlekar et al., 2022	Recurrent BC	Gaussian mixture
ACT Zhao et al. (2023)	Action-chunking transformer	Chunked action generation
DP-CNN Chi et al. (2024)	Diffusion + U-net	Continuous denoising
DP-transformer Chi et al. (2024)	Diffusion + transformer	Continuous denoising

Because FDP relies on object-centric bounding-box cues, the main baseline comparison should be interpreted together with the ablation studies. The main baseline set evaluates overall policy effectiveness, whereas reduced-model comparisons and bounding-box perturbation experiments isolate the contribution of foveated object conditioning. This design allows the main comparison to evaluate end-to-end task performance while reserving attribution of gains from object-guided conditioning to targeted ablation analyses.

### 3.2 Results and comparative analysis

#### 3.2.1 Main results

**FIGURE 6:**
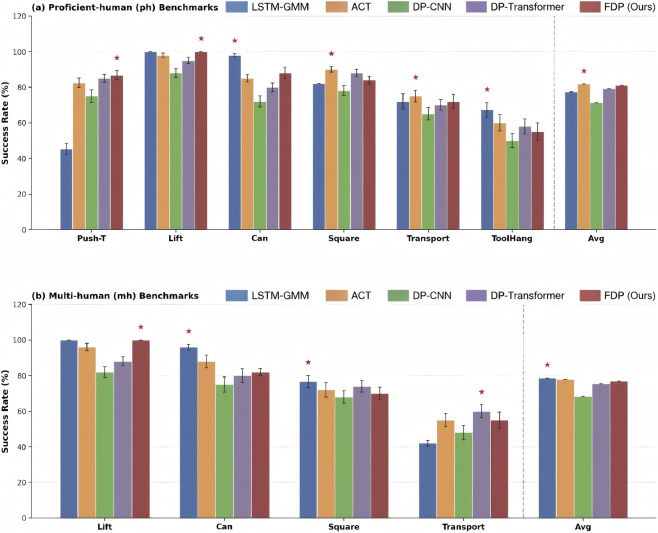
Overall comparison of FDP and baseline methods across the warehouse-relevant simulated manipulation benchmarks. **(a)** Mean success rate on proficient-human (ph) benchmarks. **(b)** Mean success rate on multi-human (mh) benchmarks.

We evaluate the proposed FDP on ten benchmark settings covering six warehouse-relevant simulated manipulation task types, including planar pushing, single-arm grasping and placement, bimanual transport, and precision insertion. Lift, Can, Square, and Transport are evaluated under both proficient-human and multi-human demonstration settings. SR is used as the primary metric and is reported as mean ± standard deviation over three random seeds. Figure 6 summarizes the overall comparison, while Tables 6, 7 report detailed results for the standard/proficient-human group and the multi-human group, respectively.

**TABLE 6:** Success rates (%) on standard/proficient-human benchmark settings.

Method	Push-T	Lift	Can	Square	Transport	ToolHang	Avg
LSTM-GMM Mandlekar et al. (2022)	45.2±3.1	100.0±0.0	98.0±0.9	82.0±0.0	72.0±4.3	67.3±4.1	77.4
ACT Zhao et al. (2023)	82.5±2.8	98.0±1.2	85.0±2.1	90.0±1.5	75.0±3.2	60.0±4.5	81.8
DP-CNN Chi et al. (2024)	75.0±3.5	88.0±2.5	72.0±3.0	78.0±2.8	65.0±3.5	50.0±4.0	71.3
DP-transformer Chi et al. (2024)	85.0±2.2	95.0±1.8	80.0±2.5	88.0±2.0	70.0±3.0	58.0±4.2	79.3
FDP (ours)	86.7±2.5	100.0±0.0	88.0±3.2	84.0±2.1	72.0±4.0	55.0±5.0	81.0

**TABLE 7:** Success rates (%) on multi-human benchmark settings.

Method	Lift	Can	Square	Transport	Avg
LSTM-GMM Mandlekar et al., 2022	100.0±0.0	96.0±1.6	76.7±3.4	42.0±1.6	78.7
ACT Zhao et al. (2023)	96.0±2.1	88.0±3.5	72.0±4.0	55.0±3.8	77.8
DP-CNN Chi et al. (2024)	82.0±3.0	75.0±4.2	68.0±3.5	48.0±4.0	68.3
DP-transformer Chi et al. (2024)	88.0±2.5	80.0±3.8	74.0±3.2	60.0±3.5	75.5
FDP (ours)	100.0±0.0	82.0±2.0	70.0±3.5	55.0±4.5	76.8

The reported comparisons are intended to assess not only task completion performance, but also the robustness of object-grounded embodied picking under visual disturbance, demonstration heterogeneity, and precision-demanding manipulation conditions.

Across the standard/proficient-human benchmark group, FDP achieves an average SR of 81.0%. This result is close to the strongest overall baseline, ACT, at 81.8%, and exceeds DP-Transformer, DP-CNN, and LSTM-GMM in average performance. The aggregate margin is moderate rather than dominant, but the task-level pattern shows that foveated conditioning provides targeted benefits in visually grounded manipulation.

FDP achieves the best performance on Push-T, ties for the best result on Lift-ph, and obtains competitive results on Can-ph and Transport-ph. The Push-T result provides direct evidence for visually grounded planar control. FDP reaches 86.7±2.5% , outperforming ACT by 4.2 percentage points and DP-Transformer by 1.7 percentage points. Lift-ph is saturated by multiple methods, with FDP and LSTM-GMM both reaching 100.0±0.0% ; therefore, this task mainly confirms learning stability rather than strongly discriminating among high-performing policies.

Can-ph provides more informative evidence for object-centered placement. FDP obtains 88.0±3.2% , ranking behind LSTM-GMM but exceeding ACT and DP-Transformer. This suggests that the proposed foveated pathway can strengthen placement-oriented manipulation by preserving target-relevant evidence during action generation. On Square-ph, FDP remains competitive but below ACT and DP-Transformer, indicating that transformer-based baselines are still strong for terminal insertion and alignment. On Transport-ph and ToolHang, FDP remains within a competitive range, although the results also show that multi-view coordination and fine manipulation continue to be challenging.

From the perspective of e-commerce warehouse fulfillment, these results suggest that FDP is most relevant to operational settings in which visual grounding and target stability are critical, such as cluttered bin picking, object placement under visual distraction, and precision-sensitive handling of packaged goods. The results do not imply that FDP universally replaces existing visuomotor policies. Rather, they show that foveated object grounding provides a practical architectural enhancement for tasks where target-object evidence must remain closely coupled with action generation.

The multi-human benchmarks introduce greater demonstration heterogeneity and therefore provide a stricter evaluation of policy robustness. FDP achieves an average SR of 76.8%, which is close to LSTM-GMM and ACT and higher than DP-Transformer and DP-CNN. This indicates that FDP retains competitive robustness under multi-human demonstrations, although it does not dominate all baselines on every task.

On Lift-mh, FDP maintains 100.0±0.0% , matching LSTM-GMM and outperforming ACT, DP-Transformer, and DP-CNN. This confirms that the object-grounded pathway remains stable when the target interaction geometry is simple and consistent across demonstrators. On Can-mh, FDP reaches 82.0±2.0% , which is lower than LSTM-GMM and ACT but higher than DP-Transformer and DP-CNN. This result suggests that the proposed method improves over diffusion-based baselines on heterogeneous placement demonstrations, while also showing that explicit object grounding alone does not fully eliminate the difficulty caused by diverse human strategies.

On Square-mh, FDP remains competitive but below LSTM-GMM, ACT, and DP-Transformer. On Transport-mh, FDP reaches 55.0±4.5% , matching ACT and ranking behind DP-Transformer. Taken together, the multi-human results indicate that FDP provides competitive robustness under demonstration diversity, especially when target interaction remains structurally identifiable. Its advantage is most evident relative to diffusion-based and CNN-based baselines, while strong behavioral and transformer-based baselines remain competitive on specific tasks.

For e-commerce warehouse fulfillment, these findings suggest that object-grounded embodied picking is valuable for maintaining task-relevant perception under heterogeneous demonstrations. However, future extensions may still require strategy-conditioned imitation learning, trajectory clustering, or hierarchical action selection to handle tasks with highly diverse manipulation styles.

Overall, the main results support three conclusions. First, FDP achieves a competitive average performance across both benchmark groups. Second, its strongest empirical contribution lies in visually grounded manipulation and object-centered placement, as reflected by the Push-T, Lift, and Can results. Third, the method improves robustness through target-conditioned action generation, but its advantage remains task-dependent rather than universal.

#### 3.2.2 Ablation experiments

We conduct ablation experiments to quantify the contribution of the main design choices in FDP. The ablations are organized around three questions: whether foveated conditioning contributes beyond the diffusion backbone, whether the method remains stable under imperfect target localization, and whether the selected temporal configuration is appropriate.

Table 8 isolates the effect of bbox-guided foveated conditioning by comparing the full FDP model with a variant that removes object-guided foveated cross-attention while retaining the diffusion backbone and non-foveated visual conditioning. The full model improves the average SR from 80.0% to 91.3% on the diagnostic ablation subset, corresponding to an 11.3 percentage point gain. This result indicates that the improvement cannot be attributed solely to the diffusion backbone; rather, the object-conditioning pathway provides a consistent contribution across the selected ablation tasks.

**TABLE 8:** Ablation on foveated cross-attention mechanism.

Configuration	Push-T	Lift-ph	Lift-mh	Can-ph	Can-mh	Avg
Full model	86.7	100.0	100.0	88.0	82.0	91.3
W/o foveated attention	75.0	84.0	82.0	84.0	75.0	80.0
Δ improvement	+11.7	+16.0	+18.0	+4.0	+7.0	+11.3

The largest improvements appear on Lift-ph and Lift-mh, where foveated attention improves performance by 16.0 and 18.0 percentage points, respectively. These results indicate that target-centered visual evidence substantially enhances execution stability when the object interaction pattern is structurally consistent. Push-T also improves by 11.7 percentage points, suggesting that object grounding benefits visually guided planar control. On Can-ph and Can-mh, FDP improves performance by 4.0 and 7.0 percentage points, respectively. Although the gains are smaller than those on Lift, they still indicate that foveated conditioning contributes positively to placement-oriented manipulation under both proficient-human and multi-human settings.

To separate the dual-stream design from the focusing modules themselves, we compare FDP with a soft-attention variant, an STN variant, and their parallel combination, as shown in Table 9. All are trained under an identical backbone, diffusion model, optimizer, and schedule on Push-T and differ only in the focusing mechanism. Under clean detection, no variant exceeds FDP, and the parallel combination does not surpass its strongest single component; the gain is therefore not produced by stacking modules. Under progressive detection failure, only FDP retains a detection-independent context stream as a fallback; at full wrong-object corruption, it holds 77.0% success, close to the no-foveation level, whereas the variants fall below 53%. The advantage of the dual-stream design thus emerges specifically under imperfect localization.

**TABLE 9:** Push-T success rate (%) under a matched backbone and training protocol.

Method	Focusing mechanism	Clean SR	F2 = 100% SR	Mean slope
No-foveation	Single context stream	75.0	75.0	0.00
STN variant	Global affine warp	80.5	48.0	3.25
Parallel (SA ‖ STN)	Concat-then-project	84.5	50.5	3.40
Soft-attention variant	Self-learned attention	85.2	52.5	3.27
FDP (ours)	Dual-stream + ROI align	**86.7**	**77.0**	**0.97**

Bold values indicate the performance improvements achieved by the proposed method over the compared methods.

The ablation results support the central design premise of FDP, separating object-centric evidence from global scene context, and injecting this evidence during diffusion denoising improves target-conditioned action generation. At the same time, the no-foveation variant remains functional, with an average SR of 80.0%. This suggests that the diffusion policy backbone already provides a strong base capability, while foveated attention acts as a complementary mechanism that improves visual grounding and execution reliability.

Table 10 evaluates the sensitivity of FDP to bounding-box quality. Perturbations are applied in image-coordinate space before normalization. Compared with clean boxes, moderate perturbation reduces the average SR from 94.0% to 89.0%, while severe perturbation further reduces it to 84.0%. The degradation is progressive rather than abrupt, indicating that FDP is sensitive to localization quality but does not collapse under imperfect target localization. This property is important for warehouse-relevant applications, where object detectors may be affected by occlusion, reflective packaging, similar neighboring items, and viewpoint variation.

**TABLE 10:** Sensitivity to bounding box annotation quality.

Bbox quality	Description	Lift-ph	Can-ph	Avg
Ground truth	Exact annotations	100.0	88.0	94.0
Noisy ( σ=5 px)	Moderate perturbation	95.0	83.0	89.0
Noisy ( σ=10 px)	Severe perturbation	88.0	80.0	84.0
No bbox	Baseline (no foveation)	84.0	74.0	79.0

The comparison with the no-bbox condition further confirms the value of target localization. Even under severe perturbation, FDP achieves an average SR of 84.0%, which remains 5.0 percentage points higher than the no-foveation variant. With clean boxes, the advantage over the no-bbox condition reaches 15.0 percentage points. The task-level pattern also shows that both Lift and Can benefit from object localization, although Can remains more sensitive to perturbation because placement tasks require more accurate spatial grounding. These results suggest that FDP does not require perfect localization to remain useful, but improved detector quality can further strengthen performance in placement-oriented warehouse manipulation.

To verify that detector tolerance is not limited to mild localization noise, we extend the bounding-box perturbation analysis into a structured detector-failure benchmark with failure modes injected at increasing severity: complete missed detection, wrong-object boxes, and partial occlusion. Under complete missed detection, FDP degrades gracefully rather than collapsing, retaining usable success because the detection-independent context stream continues to provide global scene evidence after the fovea stream loses its box input; under wrong-object boxes, the change in success rate is within sampling noise. Partial occlusion in warehouse scenes manifests primarily as detection-level box loss, the occlusion-induced bbox loss already identified in our failure-mode analysis (Table 13), and is captured by these regimes, whereas image-level pixel corruption degrades all visual methods indiscriminately, indicating that the fault-tolerance advantage of FDP is localized in the detection-conditioning pathway.

**TABLE 13:** Failure mode analysis across benchmarks.

Failure mode/Outcome	Frequency	Primary cause	Affected tasks
Precision shortfall	∼ 45%	Insufficient terminal accuracy	Square, ToolHang
Occlusion-induced bbox loss	∼ 27%	Arm or occluder blocks target	Transport, ToolHang
Grasp misalignment	∼ 18%	Initial contact geometry error	Lift, can
Timeout	∼ 10%	Slow error recovery	Transport

Table 11 examines the trade-off between temporal context and memory cost. Increasing the observation horizon from 1 to 2 improves the average SR from 88.0% to 91.6%, showing that a short temporal history helps stabilize visual interpretation and action generation. Extending the horizon from 2 to 4 yields a further improvement to 93.0%, but this 1.4 percentage point gain requires an increase in GPU memory from approximately 10 GB–14 GB. Increasing the horizon to 8 does not improve the average SR and raises memory usage to approximately 20 GB.

**TABLE 11:** Effect of observation horizon length.

$T_{o}$	Push-T	Lift-ph	Can-ph	Avg	GPU memory
1	82.0	96.0	86.0	88.0	∼ 8 GB
2 (default)	86.7	100.0	88.0	91.6	∼ 10 GB
4	87.0	100.0	92.0	93.0	∼ 14 GB
8	86.5	99.0	91.5	92.3	∼ 20 GB

These results justify the use of $T_{o}=2$ as the default setting. Although a horizon of 4 achieves the highest average SR, its improvement over the default is small relative to the additional memory cost. The default setting therefore provides a more balanced configuration for deployment-oriented embodied picking. In warehouse fulfillment, longer histories may help under temporary occlusion or delayed visual recovery, but they also reduce scalability when multiple cameras, larger batch sizes, or multiple robots are involved. The selected horizon thus reflects a practical balance between robustness and computational efficiency.

**FIGURE 7:**
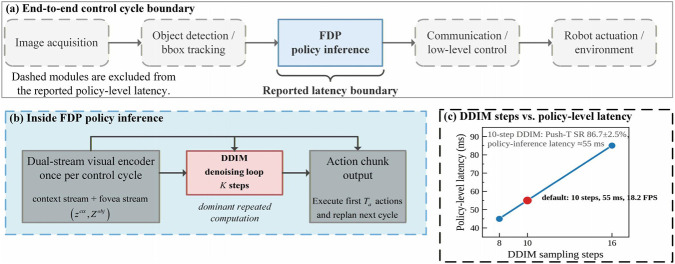
Policy-level latency boundary and DDIM sampling trade-off. **(a)** End-to-end control-cycle boundary, showing that image acquisition, object detection/bounding-box tracking, communication, low-level control, and robot actuation are excluded from the reported policy-level latency. **(b)** Computation included within FDP policy inference, comprising the dual-stream visual encoder, the repeated DDIM denoising loop, and action-chunk output. **(c)** Relationship between the number of DDIM sampling steps and policy-level inference latency; the default 10-step configuration achieves approximately 55 ms latency (18.2 FPS) and a Push-T success rate of 86.7 ± 2.5%.

Table 12 reports policy inference latency under different DDIM sampling steps. Since the main success-rate comparisons are reported in Tables 6, 7, this analysis focuses on the latency side of the efficiency trade-off. During inference, only the denoising loop is evaluated repeatedly. It is executed once per DDIM sampling step. The panorama-fovea encoder is evaluated only once per control cycle. The two streams share one ResNet-18 backbone. The context stream operates on a low-resolution feature map. The fovea stream adds only ROI Align over the N=2-3 detected boxes. Therefore, the dual-stream encoder introduces limited overhead relative to the N -step denoising loop. The number of DDIM steps is the dominant factor for inference latency. We use DDIM with 10 steps instead of the 100-step DDPM schedule. This reduces the dominant inference cost by about one order of magnitude. At this operating point, FDP achieves a Push-T success rate of 86.7±2.5% . The measured policy-inference latency is approximately 55 ms on an RTX 4090, corresponding to about 18 FPS. This result supports the low-latency feasibility of FDP at the policy level, we further visualize the latency measurement boundary in Figure 7. It does not include image acquisition, object detection, communication, or low-level control. Therefore, the results should be interpreted as evidence of policy-level low-latency feasibility rather than as a complete end-to-end warehouse-cycle-time measurement.

**TABLE 12:** Performance-latency trade-off across diffusion steps.

Sampling strategy	Steps	Latency	Deployment implication
DDIM	8	∼ 45 ms	Fastest sampling, but may reduce precision-sensitive performance
DDIM (default)	10	∼ 55 ms	Balanced setting for closed-loop action generation
DDIM	16	∼ 85 ms	Higher computational cost with limited additional benefit

#### 3.2.3 Qualitative analysis

**FIGURE 8:**
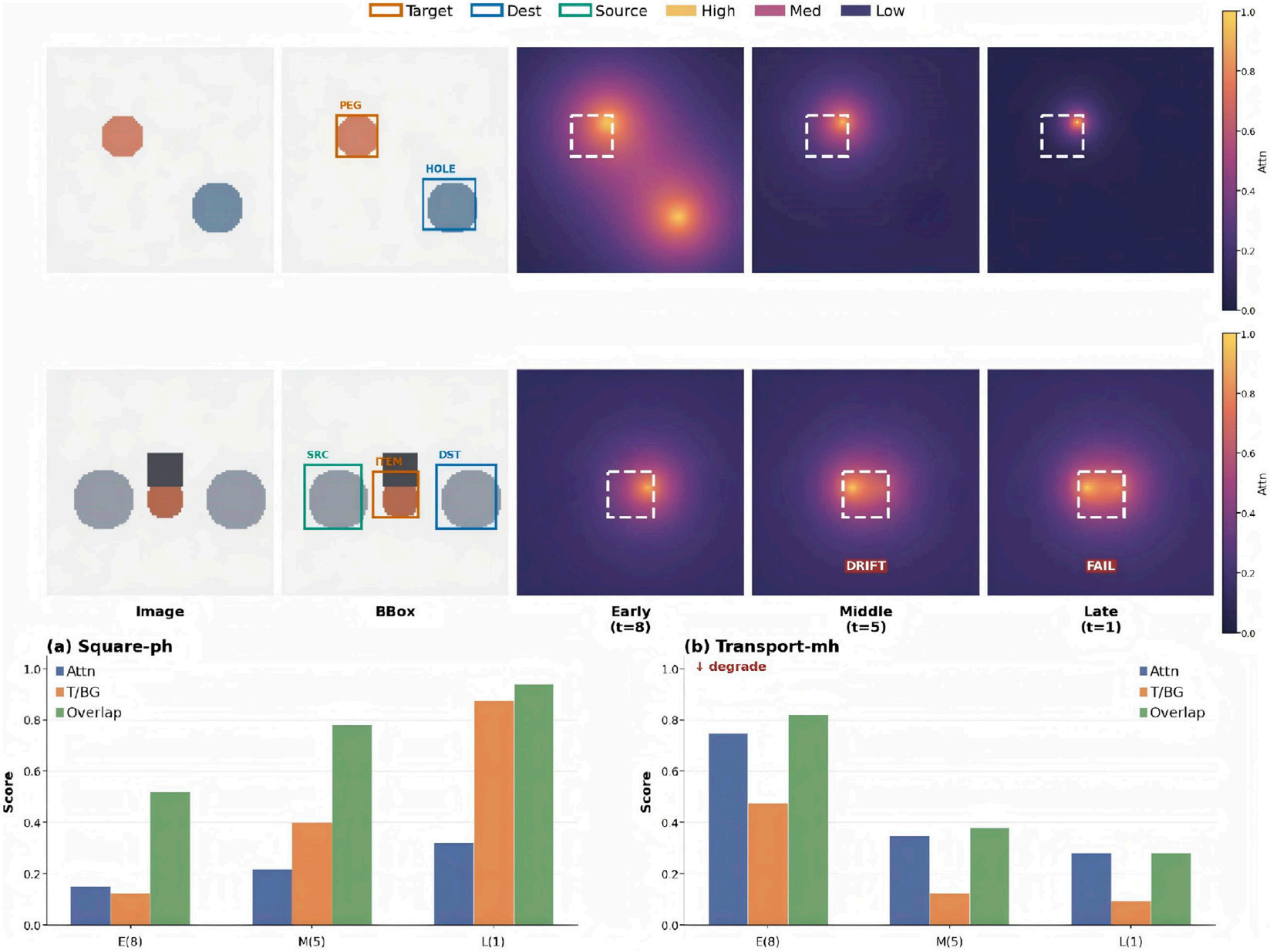
Representative attention maps during policy execution. **(a)** Successful Square-ph case, showing the input image, detected bounding boxes, and attention maps at the early (t = 8), middle (t = 5), and late (t = 1) denoising stages, together with the corresponding attention-score analysis. **(b)** Challenging Transport-mh case, showing attention drift under a more difficult multi-human manipulation setting and the corresponding degradation in attention scores. Target, destination, and source-object regions are indicated by the colored bounding boxes, while the heatmaps show the spatial attention intensity.

Figure 8 visualizes one successful Square-ph case and one challenging Transport-mh case to examine how foveated attention behaves under representative success and failure conditions.

In the successful Square case, the attention maps show a progression from coarse localization to more concentrated responses around the target interaction region. At early denoising steps, the model attends broadly to the manipulated object and target region. At intermediate steps, the response becomes more concentrated around the peg-slot interaction area. At late steps, the strongest attention is localized near the terminal contact region required for precise alignment. This temporal evolution suggests that FDP first establishes global target awareness and then progressively refines action generation using local geometric cues. Irrelevant background regions receive lower attention weights, which is consistent with the intended role of the foveated pathway in reducing distractor influence.

In the difficult case, the attention initially responds to the correct task-relevant region, but its concentration becomes less stable when the target is partially occluded or visually similar regions compete for attention. Under such conditions, the response may spread toward the occluding arm, neighboring objects, or surrounding clutter, weakening the reliability of target-conditioned action refinement. This behavior is consistent with the failure patterns summarized in Table 13, particularly occlusion-induced bbox loss and precision shortfall. Overall, the Figure 8 provides interpretability evidence that foveated attention improves target-centered visual grounding, while also revealing the conditions under which the mechanism becomes less reliable. The attention maps should be interpreted as descriptive cues rather than causal proof.

**FIGURE 9:**
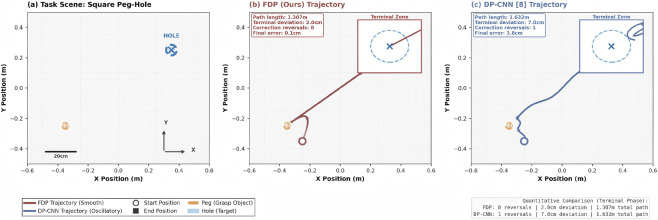
Representative trajectory comparison between FDP and the no-foveation variant. **(a)** Initial Square Peg-Hole task scene, including the starting position, peg, and target hole. **(b)** Trajectory generated by FDP, showing a smoother approach, fewer terminal reversals, and a smaller final alignment error. **(c)** Trajectory generated by the no-foveation DP-CNN variant, showing greater oscillation, more corrective reversals, and a larger terminal deviation. The inset panels enlarge the terminal alignment region.

Figure 9 compares FDP with the no-foveation variant under the same initial scene and target configuration. A precision-sensitive task such as Square is used because the benefits of stable target-conditioned control are easier to interpret in the terminal alignment phase.

The overlaid trajectories show that FDP generates a smoother approach path and more compact terminal corrections near the insertion region. Once the target is localized, the policy maintains a relatively coherent approach direction and requires fewer local adjustments before reaching the final alignment state. In contrast, the no-foveation variant exhibits larger deviations and more oscillatory behavior near the insertion point, indicating less stable terminal control and a greater reliance on repeated corrective motion. This difference is especially visible in the zoomed terminal region, where FDP shows fewer reversals and a more concentrated endpoint distribution.

This qualitative pattern complements the quantitative findings in Tables 6–8. While the numerical results demonstrate that FDP is competitive on precision-sensitive manipulation and benefits from foveated conditioning, the trajectory comparison reveals how these advantages emerge at the behavioral level. Specifically, repeatedly injecting object-centered evidence during denoising appears to stabilize motion planning near the target, resulting in smoother execution and more reliable terminal adjustment. For warehouse-oriented embodied picking, such improvements are operationally meaningful because they may reduce collision risk, item damage, and unnecessary retry behavior during fine manipulation.

We further analyze failure cases to identify remaining challenges. Table 13 categorizes common failure modes observed across failed rollouts.

Table 13 summarizes the main failure categories observed across failed rollouts. Precision shortfall is the most frequent failure mode, especially in Square and ToolHang, where small terminal errors can cause task failure even when the approach trajectory is largely correct. Occlusion-induced bbox loss occurs when the robot arm, an occluding object, or a neighboring item blocks the target region, thereby disrupting the foveated conditioning pathway. Grasp misalignment is mainly observed in Lift and Can, where initial contact geometry determines whether the object can be lifted or placed successfully. Timeout failures occur when the policy cannot recover quickly from suboptimal intermediate states, particularly in longer-horizon coordination tasks such as Transport.

These failure patterns identify several directions for further improvement. First, temporal bbox tracking and multi-view fusion could reduce sensitivity to transient occlusion. Second, hierarchical control could separate coarse approach from fine terminal manipulation, improving precision in insertion and hanging tasks. Third, recovery-aware policy learning could improve behavior after suboptimal states. These directions are directly relevant to e-commerce warehouse fulfillment, where exception recovery, retry behavior, and cycle-time stability affect operational robustness.

Taken together, the qualitative results support the quantitative analysis. FDP improves target-centered visual grounding, produces smoother trajectories in representative cases, and exhibits interpretable failure modes. The method is therefore best understood as a practical enhancement for object-grounded embodied picking under warehouse-relevant visual disturbances, rather than as a universal solution to all manipulation and demonstration-heterogeneity challenges.

### 3.3 Limitations

Despite the targeted gains and competitive performance achieved by FDP, several limitations remain. First, the method relies on bounding-box-based target-localization cues. Although the perturbation analysis suggests tolerance to moderate localization noise, performance may still degrade under detector delay, severe occlusion, or persistent mis-localization. Second, iterative denoising continues to impose non-negligible computational cost, especially on multi-camera and precision-sensitive tasks. The current DDIM-based acceleration should therefore be regarded as a practical compromise rather than a complete solution to real-time deployment. Third, the present evaluation is restricted to simulated benchmarks relevant to warehouse fulfillment. Therefore, the robustness of FDP under real-world visual drift, sensing noise, calibration error, execution uncertainty, and end-to-end warehouse cycle-time constraints remains to be verified.

These limitations indicate that the current study provides evidence for the effectiveness of object-grounded embodied picking in warehouse-relevant simulated settings, but not yet a full validation of real-world warehouse deployment. Future work should extend the evaluation to physical warehouse testbeds, integrate detector latency into end-to-end cycle-time analysis, and examine how object-grounded embodied policies interact with operational variables such as exception recovery, item damage risk, and throughput stability.

## 4 Conclusion

This study proposed a FDP for object-grounded embodied picking in e-commerce warehouse fulfillment. By separating scene-level context from target-centered evidence through a panorama–fovea dual-stream encoder and injecting object tokens into diffusion-based action generation via object-guided cross-attention, FDP strengthens the coupling between visual grounding and action refinement under cluttered and visually variable conditions. Experiments on warehouse-relevant simulated manipulation benchmarks show that FDP provides competitive and task-dependent performance rather than universal superiority, with clearer benefits in object-centered and visually grounded settings and with consistent gains over the no-foveation variant. These findings suggest that object-grounded visual conditioning is a practical design direction for improving the operational robustness of embodied picking, particularly by reducing reliance on spurious contextual cues and supporting more stable target-conditioned execution. Nevertheless, the present evidence remains limited to simulated settings, and further validation under real warehouse sensing noise, detector latency, SKU diversity, packaging variation, and end-to-end cycle-time constraints is required before deployment in e-commerce fulfillment environments.

## Data Availability

The original contributions presented in the study are included in the article/Supplementary Material, further inquiries can be directed to the corresponding author.
